# Integrated Platform for Assessing the Quality of Macromolecular Models

**DOI:** 10.1063/4.0001054

**Published:** 2025-10-27

**Authors:** Wojciech Dec, Pawel Rubach, Wladek Minor

**Affiliations:** 1 Department of Molecular Physiology and Biological Physics University of Virginia, School of Medicine, Charlottesville, Virginia, USA; 2 Department of Computational Biophysics and Bioinformatics, Jagiellonian University, Krakow, Malopolska, Poland; 3 Doctoral School of Exact and Natural Sciences, Jagiellonian University, Krakow, Malopolska, Poland; 4 Warsaw School of Economics, Warsaw, Mazowieckie, Poland

## Abstract

Macromolecular models are crucial for biomedical research, particularly in drug discovery. However, variations in model quality can impact the reliability of downstream analyses [1, 2]. To advance the evaluation of macromolecular structures, we developed an integrated system for the rapid exploration and comparison of protein structure models derived from cryoEM and crystallography experiments. Our platform seamlessly incorporates multiple resources, including RCSB PDB [3], PDBe [4], PDBj [5], UniProt [6], PDB-REDO [7, 8], EMDB [9], and AlphaFold DB [10]. Currently, the system operates within the PyMOL 3.1 ecosystem, providing an efficient and streamlined workflow for structural analysis.
